# Effect of sex on the APOE4-aging interaction in the white matter microstructure of cognitively normal older adults using diffusion-tensor MRI with orthogonal-tensor decomposition (DT-DOME)

**DOI:** 10.3389/fnins.2023.1049609

**Published:** 2023-02-22

**Authors:** Patcharaporn Srisaikaew, Jordan A. Chad, Pasuk Mahakkanukrauh, Nicole D. Anderson, J. Jean Chen

**Affiliations:** ^1^Ph.D. Program in Anatomy, Faculty of Medicine, Chiang Mai University, Chiang Mai, Thailand; ^2^Department of Anatomy, Faculty of Medicine, Chiang Mai University, Chiang Mai, Thailand; ^3^Rotman Research Institute, Baycrest Health Sciences, Toronto, ON, Canada; ^4^Department of Medical Biophysics, University of Toronto, Toronto, ON, Canada; ^5^Excellence in Osteology Research and Training Center, Chiang Mai University, Chiang Mai, Thailand; ^6^Department of Psychology and Psychiatry, University of Toronto, Toronto, ON, Canada; ^7^Institute of Biomedical Engineering, University of Toronto, Toronto, ON, Canada

**Keywords:** apolipoprotein E4 gene, cognitively normal older adults, diffusion tensor imaging (DTI), tensor decomposition, white matter (WM), sex effects, mode of anisotropy (MO), norm of anisotropy (NA)

## Abstract

The influence of the apolipoprotein E ε4 allele (APOE4) on brain microstructure of cognitively normal older adults remains incompletely understood, in part due to heterogeneity within study populations. In this study, we examined white-matter microstructural integrity in cognitively normal older adults as a function of APOE4 carrier status using conventional diffusion-tensor imaging (DTI) and the novel orthogonal-tensor decomposition (DT-DOME), accounting for the effects of age and sex. Age associations with white-matter microstructure did not significantly depend on APOE4 status, but did differ between sexes, emphasizing the importance of accounting for sex differences in APOE research. Moreover, we found the DT-DOME to be more sensitive than conventional DTI metrics to such age-related and sex effects, especially in crossing WM fiber regions, and suggest their use in further investigation of white matter microstructure across the life span in health and disease.

## Highlights

- The APOE4 association with age-related WM changes in older adults differs by sex.
- Aging is similarly associated with WM integrity in both APOE4+and APOE4−.
- DT-DOME parameters are more sensitive to age and sex than conventional DTI metrics.

## 1. Introduction

Cerebral white matter (WM) tracts undergo various neurodegenerative changes in normal aging. The apolipoprotein E (APOE) gene is a known Alzheimer’s disease (AD) risk factor that may alter the course of WM degeneration. The APOE gene is polymorphic and consists of three major isoforms–epsilon2 allele (ε2), epsilon3 allele (ε3), and epsilon4 allele (ε4), each associated differently with AD risk. The APOE ε3 allele is the most common and is considered a neutral allele, neither decreasing nor increasing AD risk. The APOE ε2 allele is protective against AD but is relatively rare, while the APOE ε4 allele (APOE4) has been long known as the primary genetic risk factor for sporadic AD (Martins et al., 2005; Zhao and Wu, 2016). Having one or two copies of APOE4, therefore, increases the risk of developing AD in aging (Liu et al., 2015; Paraskevaidi et al., 2017; Fernandez et al., 2019). However, the interaction between APOE4 and brain microstructure in the aging process before AD onset is still not fully understood. A summary of research to date is provided in Supplementary Table 1.

Diffusion tensor imaging (DTI) has been widely used to study *in vivo* WM microstructure in the human brain (Liu et al., 2009). Conventional DTI metrics include fractional anisotropy (FA) and mean diffusivity (MD), and MD can be separated into axial diffusivity (AxD) and radial diffusivity (RD). Numerous DTI studies have been conducted to investigate the effect of APOE4 on healthy adult aging (Honea et al., 2009; Adluru et al., 2014; Dell’Acqua et al., 2015; Wang et al., 2015; Cai et al., 2017; Cavedo et al., 2017; Operto et al., 2018). The majority of DTI studies in healthy older adults have reported lower FA and higher diffusivities in APOE4 carriers than non-carriers, with multiple WM tracts implicated, such as the cingulum bundle, corpus callosum, and superior longitudinal fasciculus. These findings suggest that APOE4 is related to WM fiber degeneration (e.g., axonal degeneration and loss of myelin sheath density; (Nierenberg et al., 2005; Persson et al., 2006; Honea et al., 2009; Heise et al., 2011; Salminen et al., 2013; Adluru et al., 2014; Cavedo et al., 2017; Slattery et al., 2017). Saddiki et al. (2020) found that the impact of APOE4 on brain aging was most pronounced in individuals aged 65 to 70 years old. Females are more likely to carry APOE4 than males (Bretsky et al., 1999; Mortensen and Høgh, 2001; Beydoun et al., 2012; Altmann et al., 2014), but the role of sex in APOE4 effects on WM microstructure remains under-investigated (see Supplementary Table 2 for a summary of literature on sex and APOE4-related effect in aging and dementia). In fact, as can be seen in Supplementary Tables 1, 2, most relevant research studies to date have different male-to-female ratios in their carrier and non-carrier groups.

While our knowledge of APOE4 interactions with brain microstructure is almost solely based on DTI, there are a number of challenges to interpreting conventional DTI findings in general. One challenge is the presence of voxels with crossing fibers or other complex fiber architectures (Basser et al., 2000). The vast majority of WM voxels contain more than one fiber bundle, limiting the interpretation of FA as a measure of single-fiber integrity (Jeurissen et al., 2013; Schilling et al., 2017). For example, sporadic increases in FA with age have been observed in the corona radiata and internal capsule thought to be due to selective degeneration of a secondary fiber tract, which results in increased directionality along the primary direction (de Groot et al., 2016; Chad et al., 2021). Whether fiber degeneration manifests as a decrease or increase in FA, therefore, depends on the precise fiber architecture. Another challenge of interpreting DTI studies is that FA is by definition conflated with MD, such that an increase in MD results in a decrease in FA even in the absence of fiber degeneration. This is especially problematic in the corona radiata and internal capsule, where the increase in MD can cancel out the increase in FA, which explains why FA is not very sensitive to age in these regions.

We recently introduced the use of a “diffusion tensor decomposition based on orthogonal moments of the eigenvalues” (DT-DOME) to produce tensor-shape metrics that are independent of MD–mode of anisotropy (MO) and norm of anisotropy (NA) (Chad et al., 2021). Unlike FA, MO, and NA are not confounded by MD, and together with MD provide a full orthogonal decomposition of the diffusion tensor and can better characterize microstructural variations in aging. Specifically, higher NA indicates greater anisotropy and greater MO indicates a more linear tensor shape (Ennis and Kindlmann, 2006). Our recent work suggests these metrics provide superior sensitivity over conventional ones (e.g., FA) to neurodegeneration in regions of expected selective degeneration of secondary fibers (Chad et al., 2021).

In the present study, we aimed to investigate the impact of APOE4 carrier status on age differences in WM microstructure in cognitively normal adults, with a special focus on the role of sex differences. In doing so, we also leverage the DT-DOME – which is easily implementable for any DTI data–to uncover effects that may be undetectable by conventional DTI.

## 2. Materials and methods

### 2.1. Participants

**FIGURE 1:**
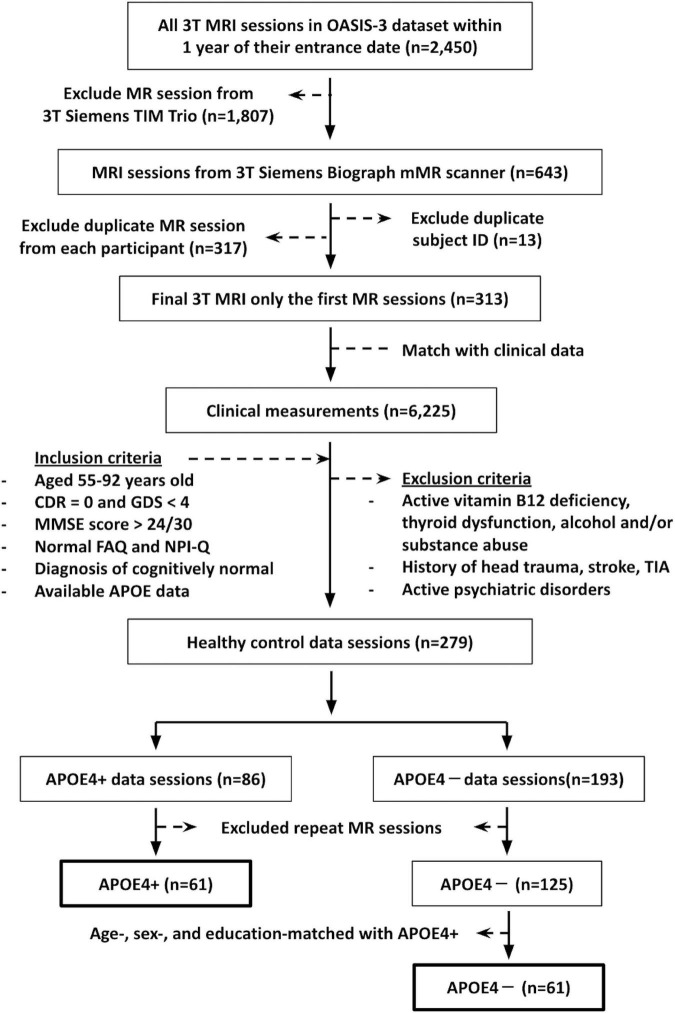
The summary flowchart of the recruitment of participants from the open-access series of imaging studies (OASIS-3) database. CDR, Clinical Dementia Rating; GDS, Geriatric Depression Scale score; MMSE, Mini-Mental State Exam score; FAQ, Functional Activities Questionnaires; NPI-Q, The Neuropsychiatric Inventory–Questionnaire; TIA, transient ischemic attack.

This study involved cognitively normal participants aged 55–92 years old, whose data were accessed from the open-access series of imaging studies (OASIS-3) database.^1^ Inclusion criteria included: (1) DTI data from a 3T Siemens BioGraph mMR PET-MR system (the platform with the most scans available); (2) scan date within 1 year of their study entrance date; (3) available APOE data; (4) a Clinical Dementia Rating (CDR) of zero (Morris, 1993); (5) a Geriatric Depression Scale score of <4 (Sheikh and Yesavage, 1986); (6) a Mini-Mental State Exam (MMSE) score of >24 (Creavin et al., 2016); (7) normal Functional Activities Questionnaires (FAQ) results (Pfeffer et al., 1982), (8) normal behavioral assessment (The Neuropsychiatric Inventory–Questionnaire, NPI-Q) (Kaufer et al., 2000); and (9) diagnosis of cognitively normal by a physician. Participants were excluded from our study if they reported active vitamin B12 deficiency, active thyroid dysfunction, active alcohol and/or substance abuse, history of head trauma, history of stroke and/or transient ischemic attacks, or active psychiatric disorders. This yielded 61 APOE carrier (APOE4+) and 125 non-carrier (APOE4−) participants. To avoid unequal sample sizes, 61 non-carrier participants were chosen for analysis, such that the groups were matched on age, sex, and education. The summary flowchart of the participant’s recruitment is shown in Figure 1.

### 2.2. Description of the dataset

Diffusion-weighted MRI (dMRI) data were acquired on 3T Siemens BioGraph mMR PET-MR system equipped with a 16-channel head coil with b of 0 (1 volume per acquisition) and 1,000 s/mm^2^ with 65 directions, at TR = 11 s, TE = 87 ms, FOV = 240 mm, matrix size = 96 × 96 × 64, and voxel size = 2.5 mm× 2.5 mm× 2.5 mm. We used the first MRI session for each participant in this longitudinal database.

Apolipoprotein E ε4 allele genotyping information, MRI data, and dMRI processing details from the OASIS-3 dataset can be found in the study of LaMontagne et al. (2019).

### 2.3. Pre-processing

All dMRI data were denoised with the Marchenko--Pastur distribution principal-component analysis implemented in ‘‘dwidenoise’’ in MRtrix release 3.0.2^2^ (Tournier et al., 2019). The first volume (*b* = 0 s/mm^2^) was used to generate a binary brain mask *via* a combination of FSL’s Brain Extraction Tool (FSL bet) (Smith, 2002) and MRtrix’s dwi2mask (Dhollander and Connelly, 2016). Then, FSL’s DTIFIT was used to fit the diffusion tensor to each voxel to yield voxel-wise maps of FA, MD, AxD, RD, MO (Ennis and Kindlmann, 2006), as well as the 1st, 2nd, and 3rd eigenvalues (L1, L2, and L3) (Behrens et al., 2003). To obtain the full set of DT-DOME metrics (MD, NA, MO), NA was computed as three times the standard deviation of L1, L2, and L3 maps using in-house code (Ennis and Kindlmann, 2006; Chad et al., 2021). In this study, FA, MD, AxD, and RD are considered conventional DTI metrics, and MO and NA are novel DT-DOME metrics of tensor shape. The latter have the characteristic of quantifying anisotropy without being conflated with MD (Ennis and Kindlmann, 2006; Chad et al., 2021).

### 2.4. Statistical analysis

All statistical analyses on the group demographics were performed with IBM-SPSS version 26 (IBM Corp., Armonk, NY, USA). Independent *t*-tests were used to compare age, education level, and scores on the Mini-Mental State Exam (MMSE) between APOE4+ and APOE4− groups. A chi-square test was used to compare the proportion of males and females between the APOE4+ and APOE4− groups. Pearson correlation coefficient was used to explore the correlation amongst age, education, MMSE score, DTI and DT-DOME parameters across APOE4+ and APOE4− groups. All *p*-values were corrected for multiple comparisons based on false discovery rate (FDR) at *p* < 0.05. The level of statistical significance was set at α < 0.05.

To assess group differences and age effects in the DTI metrics, tract-based spatial statistics (TBSS) (Smith et al., 2006) was used. The mean FA skeleton was produced at a threshold of FA ≥0.2 to exclude voxels containing peripheral structures and partial-volume effects with gray matter (GM) and cerebrospinal fluid (CSF). Non-linear registration to the FMRIB58_FA space was applied to align the individual FA maps into the MNI152 standard space. General linear model (GLM)^3^ was used to assess the influence of the APOE4 status, age, and sex on DTI parameters. Design matrix (.mat) and contrast (.con) files were created and used specifically to assess the following analyses:

1. Effect of age:
  1.1. Age-related effects on the DTI metrics in the APOE4+ and the APOE−groups, each using a linear regression;
2. Effect of APOE4 status:
  2.1. The overall differences in DTI metrics between APOE4+ and APOE4− groups, using a one-way Analysis of Variance (ANOVA), with no covariates;
3. Effect of sex:
  3.1. Differences in DTI metrics between males and females, controlling for APOE4 status, using a one-way ANOVA with a categorical covariate (carrier status);
  3.2. Differences in age-related effects on DTI metrics between males and females, using a one-way ANCOVA, with age as a covariate;
4. Sex-APOE4 status interactions:
  4.1. Differences in DTI metrics between APOE4+ and APOE4− groups, controlling for sex, using a one-way ANOVA with a categorical covariate (sex);
  4.2. Sex-APOE4 interactions on the DTI metrics, using two-way ANOVA, with sex and carrier status as covariates.

Skeletonized DTI maps (FA, MD, AxD, RD, MO, and NA) of all participants underwent these statistical analyses with correction for multiple comparisons using FSL’s randomize threshold-free cluster enhancement (with 5,000 permutations at *p* < 0.05) (Nichols and Holmes, 2002; Winkler et al., 2014). In addition, age-related differences in DTI metrics per year were calculated in each voxel using “mri_glmfit” at *p* < 0.05 as implemented in FreeSurfer (Fischl, 2012).

We also performed region-of-interest (ROI)-based statistical analyses to complement the voxelwise analyses. The ROIs were determined based on the JHU ICBM-DTI-81 WM Labels Atlas (Wakana et al., 2004; Mori et al., 2008). To assess ROI-wise effects of APOE4 carrier status and sex on DTI metrics, SPSS was used to perform the statistical tests described above. Multiple comparisons correction was performed based on false discovery rate (FDR) using MATLAB (mafdr), producing adjusted *p*-values that were thresholded at *p* < 0.05 (Benjamini and Hochberg, 1995; Storey, 2002).

## 3. Results

One APOE4+ participant with ε2/ε4 genotype was excluded due to outlier values in diffusion data (an abnormally low RD value when averaged across the WM skeleton–more than 2 standard deviation (SD) from the group mean), resulting in final cohorts of 60 APOE4+ and 61 APOE4−.

### 3.1. Demographics and allele status

Demographic and genotyping data of the APOE4+ and APOE4− groups is provided in Table 1. There were no significant differences in age, sex, education level, or MMSE score between groups. The APOE4 + group mostly consisted of the ε3/ε4 genotype (76.67%), and most of the non-carrier group had the ε3/ε3 genotype (78.69%). There were more females than males in both APOE4+ and APOE4− groups (51.67 and 59.02%, respectively). Supplementary Figure 1 shows ROI-wise Pearson correlation coefficient (r) amongst age, education, MMSE score, DTI and DT-DOME metrics across APOE4+ and APOE4− groups. In general, diffusivity measures are inversely correlated with anisotropy measures, the latter of which are positively correlated among themselves. However, anti-correlations are also observed among NA, MO, and FA.

**TABLE 1:** Summary of participants’ demographics and Mini-Mental State Examination scores (MMSE) for APOE4 carriers (APOE4+) and non-carriers (APOE4−) and for males and females.

Variables	Group (Mean ± SD)		Group comparison		Sex (Mean ± SD)		Group comparison	
	APOE4+	APOE4−	*t*	*P*	Males	Females	*t*	*P*
Subjects (*n*)	60	61	–	–	54	67	–	–
Age (years)	70.98 ± 7.13	70.90 ± 7.05	0.063	0.950	70.80 ± 6.99	71.06 ± 7.17	0.203	0.839
Education (years)	16.27 ± 2.50	16.26 ± 2.32	0.010	0.992	16.74 ± 2.14	15.09 ± 2.55	−1.906	0.059
Sex (M: F)	29:31 (0.94M:1F)	25:36 (0.69M:1F)	0.661	0.416	–	–	–	–
MMSE score	29.03 ± 1.09 (27–30)	28.95 ± 1.26 (26–30)	0.386	0.700	29.07 ± 1.15	28.89 ± 1.21	0.865	0.389
**APOE genotypes**								
ε3/ε4 (*n*, %)	46 (76.67%)	–	–	–	24 (44.44%)	22 (32.84%)	–	–
ε4/ε4 (*n*, %)	8 (13.33%)	–	–	–	3 (5.56%)	5 (7.46%)	–	–
ε2/ε4 (*n*, %)	6 (10%)	–	–	–	2 (3.71%)	4 (5.97%)	–	–
ε3/ε3 (*n*, %)	–	48 (78.69%)	–	–	17 (31.48%)	31 (46.27%)	–	–
ε2/ε3 (*n*, %)	–	12 (19.67%)	–	–	7 (12.96%)	5 (7.46%)	–	–
ε2/ε2 (*n*, %)	–	1 (1.64%)	–	–	1 (1.85%)	–	–	–

APOE4+ included ε3/ε4, ε4/ε4, and ε2/ε4 genotypes. APOE4− included ε3/ε3, ε2/ε3, and ε2/ε2 genotypes. Epsilon2 allele, ε2; epsilon3 allele, ε3; and epsilon4 allele, ε4. The sex ratio is listed in the number of males versus females (M: F) and as the ratio (number of males per female).

### 3.2. Effect of age: Voxel-wise analysis

**FIGURE 2:**
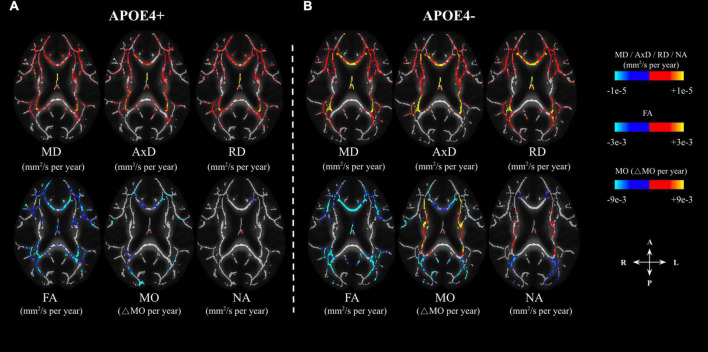
Age-related differences in DTI metrics (FA, MD, AxD, RD) and DT-DOME metrics (MO, and NA) in APOE carriers (APOE4+) and non-carriers (APOE4–) (**A,B**, respectively). The effects are shown in units of differences per year in the axial plane. The color bar shows negative (marked in blue-cyan) and positive (marked in red-yellow) change-per-year values, and only regions with statistically significant age associations (at *p* < 0.05) are shown. FA, fractional anisotropy; MD, mean diffusivity; AxD, axial diffusivity; RD, radial diffusivity; MO, mode of anisotropy; NA, norm of anisotropy.

**FIGURE 3:**
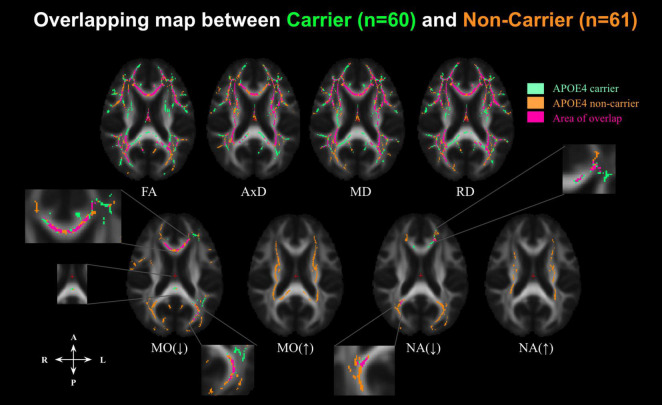
Significant associations between DTI metrics and age across the whole-brain WM in cognitively normal APOE carriers (APOE4+, indicated in green) and APOE4 non-carriers (APOE4–, indicated in orange), along with overlaps between the groups (indicated in pink) at *p* < 0.05. Aging is associated with decreasing FA and increasing diffusivity (AxD, RD, and MD). However, MO and NA have bidirectional associations with age. MO(↓) indicates where MO is negatively associated with age; MO(↑) where MO is positively associated with age; NA(↓) where NA is negatively associated with age, and NA(↑) where NA is positively associated with age. FA, fractional anisotropy; MD, mean diffusivity; AxD, axial diffusivity; RD, radial diffusivity; MO, mode of anisotropy; NA, norm of anisotropy.

In the voxel-wise analysis, we found that the age-related effects may manifest differently depending on APOE4 status (Analysis 1.1). The age-effect sizes (variations per year) associated with various parameters are shown in Figure 2, with the effect sizes highlighted only in regions with significant age associations. The APOE4− group appeared to demonstrate stronger age associations judging by the color maps (Analysis 1.1, Figures 2A, B), in addition to exhibiting positive age associations in NA and MO in the internal-capsule region (shown in orange). The similarities and differences between the significant age effects seen in the APOE4+ and APOE4− groups (Figure 2) are summarized in Figure 3. Irrespective of carrier status, widespread significant positive age associations were found in MD, AxD, and RD, while significant negative associations were found in FA, MO, and NA in both groups at *p* < 0.05. In both groups, negative age-associations in FA along with positive age-associations in MD, AxD, and RD coincided, notably in the fornix, cingulum bundle, and corpus callosum. Likewise, in both groups, negative age-associations in both MO and NA were found in the genu and body of the corpus callosum and anterior and posterior corona radiata. Interestingly, while the APOE4 − group is associated with positive age-associations with MO and NA in both anterior and posterior WM (in the splenium of the corpus callosum, superior longitudinal fasciculus, internal capsule, and superior corona radiata), the APOE4+ group was not. Overall, the differences in age-related effects between the groups were mostly seen in the posterior WM regions including the splenium of the corpus callosum and posterior thalamic radiation.

To further condense the results, overlapping and disjoint patterns between the APOE4+ and APOE4− groups in Figure 3 are summarized by the forest plots in Supplementary Figure 2. Significant negative age associations of FA overlap with positive age associations of MD, AxD, and RD in a large set of regions, including the fornix, cingulum bundle, corpus callosum, superior longitudinal fasciculus, inferior longitudinal fasciculus, internal capsule, external capsule, corona radiata, and posterior thalamic radiation regions in both groups. As shown in Supplementary Figure 2, significant negative age associations in MO and NA were evident in both groups in the genu and body of the corpus callosum and anterior and posterior corona radiata regions. However, both MO and NA exhibited significant positive age associations in the splenium of corpus callosum, superior longitudinal fasciculus, internal capsule, and superior corona radiata regions, but only in the APOE4− group.

### 3.3. Effect of APOE4 carriers status (Analysis 2.1)

We performed ROI-based statistical analyses, limited to regions showing significant age-related effects in the voxel-wise analyses (as seen in Figures 2, 3). The ROI-based group comparison (between the APOE4+ and APOE4− groups) showed that FA in the body and column of the fornix was significantly lower in APOE4− than in the APOE4+ group (adjusted *p* = 0.046). MD, AxD, and RD in the body and column of the fornix (adjusted *p* = 0.039, 0.049 and 0.039, respectively), corona radiata (adjusted *p* = 0.039, 0.039, and 0.039, respectively), and superior longitudinal fasciculus (adjusted *p* = 0.008, 0.008, and 0.039, respectively) were significantly higher in APOE4− than the APOE4+ group. In the corpus callosum, only AxD was significantly higher in the APOE4− than the APOE4+ group (adjusted *p* = 0.046). No significant difference in these conventional DTI metrics was found between groups in the cingulum, posterior thalamic radiation or internal and external capsules (see Table 2).

**TABLE 2:** Summary of statistical significance of the group comparisons among DTI metrics in various white matter tracts between APOE4 carrier (APOE4+) and non-carrier (APOE4−) groups.

WM tracts	Conventional DTI metrics				DTI metrics independent of MD	
	FA	MD	AxD	RD	MO	NA
**Fx_w**	0.107	0.128	0.185	0.108	**0.049^>^**	0.108
**Fx_BC**	**0.046^>^**	**0.039^<^**	**0.049^<^**	**0.039^<^**	0.142	0.145
**CC**	0.191	0.100	**0.046^<^**	0.108	0.202	0.163
**Cg**	0.191	0.229	0.142	0.272	0.191	0.191
**CR**	0.128	**0.039^<^**	**0.039^<^**	**0.039^<^**	0.185	0.191
**PTR**	0.202	0.229	0.235	0.223	0.222	0.272
**IC**	0.191	0.191	0.191	0.202	0.167	0.167
**EC**	0.228	0.191	0.265	0.185	0.224	0.228
**SLF**	0.202	**0.008^<^**	**0.008^<^**	**0.039^<^**	0.191	0.163
**ILF**	0.229	0.128	0.061	0.167	0.222	0.108

The values are corrected for multiple comparisons (i.e., adjusted *p*-values). The black less-than symbol (^<^) indicates the metric is lower in APOE4+ than the APOE4−. The black greater-than symbol (^>^) indicates the metric is greater in APOE4+ than the APOE4−. Fx_w, the whole tract of the fornix; Fx_BC, the body and column of the fornix; CC, corpus callosum; Cg, cingulum bundle; CR, corona radiata; PTR, posterior thalamic radiation; IC, internal capsule; EC, external capsule; SLF, superior longitudinal fasciculus; ILF, inferior longitudinal fasciculus; FA, fractional anisotropy; MD, mean diffusivity; AxD, axial diffusivity; RD, radial diffusivity; MO, mode of anisotropy; NA, norm of anisotropy. Significant values are bolded.

The group comparison of the DT-DOME metrics (MO and NA) revealed significantly lower MO in the APOE4− group (*p* = 0.049; see Table 2) when averaged across the whole fornix tract. However, no significant difference between groups was found in those showing significant group differences in FA and MD listed earlier.

### 3.4. Effect of sex

**FIGURE 4:**
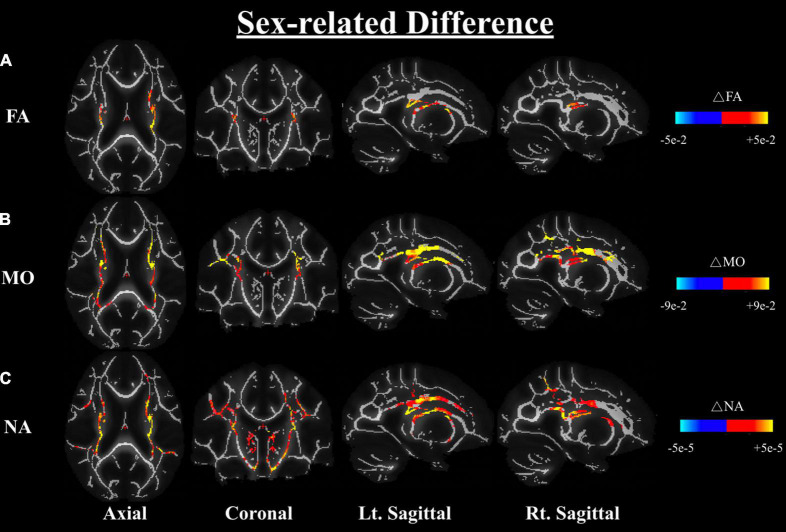
Differences in FA **(A)**, MO **(B)**, and NA **(C)** between males and females based on a one-way ANOVA while controlling the APOE4 status (Analysis 3.1). Differences are shown in the axial, coronal, and left and right sagittal planes. The color bars indicate significant sex differences (at *p* < 0.05) in FA, MO, and NA whereby red-yellow means metrics have higher values in males than females, notably in the corona radiata and the internal capsule regions. FA, fractional anisotropy; MO, mode of anisotropy; NA, norm of anisotropy; ANOVA, Analysis of Variance.

One-way ANOVA of the carrier-sex interactions between males and females on any DTI or DT-DOME metric, showed that the FA, MO, and NA are greater in males than females across the WM, most notably in the corona radiata and internal capsule (at *p* < 0.05), when controlling for APOE4 status (Analysis 3.1). No significant sex-related difference was found in MD, AxD or RD as shown in Figure 4. Moreover, the one-way ANCOVA (Analysis 3.2) revealed no significant sex difference in age-related effects in any of the parameters.

### 3.5. Sex-APOE4 status interactions

**FIGURE 5:**
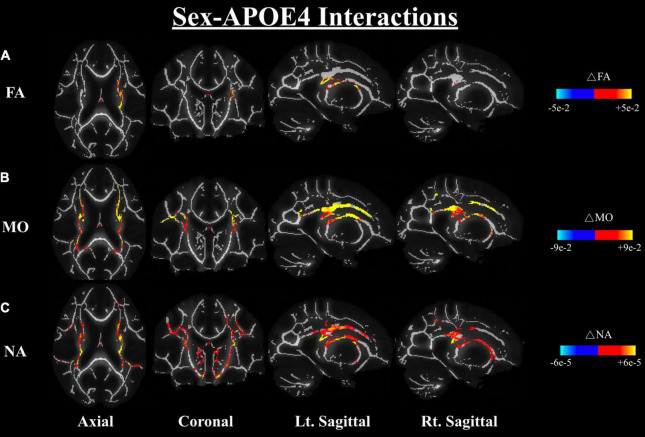
Sex-APOE4 interactions in DTI metrics were assessed using two-way ANOVA (Analysis 4.2). The significant sex difference in FA **(A)**, MO **(B)**, and NA **(C)** were mostably found in corona radiata and internal capsule at *p* < 0.05, as shown in the axial, coronal, left, and right sagittal planes. In all cases, the color bar represents differences in the metrics, whereby yellow-red indicates where APOE4+ men have higher metrics than APOE4+ women. FA, fractional anisotropy; MO, mode of anisotropy; NA, norm of anisotropy; ANOVA, Analysis of Variance.

We further investigated the differences in dMRI metrics between the APOE4+ and APOE4− groups while controlling for sex; the results also showed no significant difference between groups in any of the dMRI metrics (both conventional metrics and DT-DOME metrics) (Analysis 4.1). Furthermore, when we assessed the sex-APOE4 interactions on the DTI metrics using two-way ANOVA with both sex and carrier status as covariates (Analysis 4.2), stronger sex differences were found amongst the APOE4 + group in FA, MO, and NA, most notably in corona radiata and internal capsule at *p* < 0.05. No significant sex-related difference was found in MD, AxD, or RD (Figure 5). This is consistent with the results from Analysis 2.1 which suggests that the sex difference may surpass the APOE4 effect in the present study.

### 3.6. Conventional vs. DT-DOME metrics

The age-related effects detected by conventional DTI exhibit 100% ROI overlap between the APOE4+ and APOE4− groups, as shown in Figure 3. On the other hand, the DT-DOME metrics revealed differences between the APOE4+ and APOE4− groups–an increase of MO and NA with age was evident, but only in the APOE4− group (see Figures 2A, B).

The similarities and differences between age-related effects in the conventional and new DTI metrics are shown in Supplementary Figure 3. The forest plots show the widespread significant negative age-associations in FA, MO, and NA in the APOE4+ and in the APOE4− groups across numerous white matter tracts, including the cingulum bundle, the internal capsule, the external capsule, the genu and body of the corpus callosum, posterior thalamic radiations and anterior and superior corona radiata. In the APOE4− group, the negative associations of MO and NA with age fully coincided with those in FA, while the positive age-association with MO and NA (in the splenium of the corpus callosum, superior longitudinal fasciculus, internal capsule, and superior corona radiata regions) were not observed for FA. In the APOE4+ group, FA and MO yield more similar results with each other than they do with NA, and this is demonstrated in the correlation matrix as well (Supplementary Figure 1). Moreover, the differences in age-related effects between FA and NA/MO are mostly seen in areas of crossing fibers such as the fornix, splenium of the corpus callosum, internal capsule, external capsule, and posterior thalamic radiation.

## 4. Discussion

In this study, we observed age associations in DTI metrics across the WM that differed by APOE4 status. In addition, we found sex differences in diffusion anisotropy (not diffusivity) but only when controlling for APOE4 status. Lastly, we also found significant sex-APOE4 interactions in diffusion anisotropy (FA, MO, and NA), indicating that these sex differences are enhanced by APOE4+ status. Taken together, our results suggest that sex differences may contribute substantially to the differences generally observed between APOE4+ and APOE4−, as well as in age-related effects in these two groups.

### 4.1. The effect of APOE4 status

It has long been known that APOE4 is a genetic risk factor for sporadic AD and that it contributes to the accelerated age-related breakdown of the myelin sheath (Abondio et al., 2019). Those with at least one copy of APOE4 are at a higher risk of developing AD compared to non-carriers. Damage to the myelin sheath is reflected in reduced FA and increased diffusivity (MD), which may reflect the progressive destruction of axons (Ryan et al., 2011). It should be mentioned that APOE4 is associated with altered brain function in both patients with neurodegenerative disease (i.e., AD) (Cosentino et al., 2008) as well as in cognitively healthy participants (Bondi et al., 2005).

Previous DTI studies have found a reduction in FA along with increased diffusivities in homozygous APOE4+ compared to APOE4− adults, most notably in the cingulum bundle, corpus callosum, superior and inferior longitudinal fasciculus (Cavedo et al., 2017; Operto et al., 2018). However, in the present study, we found no significant difference between carrier and non-carrier groups using either conventional metrics or the novel DTI metrics, possibly due to the high inter-subject heterogeneity in the APOE4+ group. Indeed, previous studies involving heterozygous APOE4 (ε3/ε4) carriers also failed to show significant differences in WM integrity between healthy APOE4+ and APOE4− in younger (Dell’Acqua et al., 2015) and older adults (Wang et al., 2015). Thus, the literature lacks consensus about the effect of APOE4 on WM microstructure. Our data suggest that sex may be a main factor driving this inconsistency.

### 4.2. The effect of APOE4 on the age-related decline in WM integrity

We found significant associations of all metrics (i.e., FA, MD, AxD, RD, MO, and NA) with age across the WM, including association fibers (cingulum bundle, superior and inferior longitudinal fasciculus, and fornix), projection fibers (internal and external capsule, corona radiata, and posterior thalamic radiation), as well as commissural fibers (corpus callosum and fornix). Note that the fornix can be considered as both an association fiber (connecting limbic structures in the same hemisphere) and a commissural fiber (connecting bilateral hippocampi *via* the hippocampal commissural or the commissural of the fornix). Although the APOE4+ and APOE4− groups were age-matched, they had different age-related effects, which were most notable in the posterior WM regions, especially in the splenium of the corpus callosum and posterior thalamic radiation (Figure 1).

Surprisingly, in the present study, the APOE4+ group exhibited weaker age-related effects than the APOE4− group, especially in regions without prominent fiber crossings. Given that each APOE isoform has been shown to confer differential susceptibility to diseases, especially to AD, one possible explanation for the weaker age-related effects in our carriers group is the APOE4+ participants seem to be healthier than the APOE4− group. We did not have access to data on OASIS-3 participants’ lifestyle data, but it is possible that the APOE4+ group in our study led healthier lifestyles than the APOE group, allowing the former to participate in the fairly demanding OASIS-3 study. Indeed, a lifestyle intervention that included physical exercise, cognitive training, and social activity (Kivipelto et al., 2013) has been known to result in improved cognitive performance among older adults who carried one or two copies of the ε4 allele, as much as it did among non-carriers (Solomon et al., 2018).

Moreover, the high frequency of the neutral APOE allele (ε3/ε4) which accounted for 76.67% of the APOE4+ when compared to the homozygous risk factor APOE allele (ε4/ε4, 13.33%), and the protective APOE allele (ε2/ε4, 10.00%) may also explain why the age effect is stronger in our APOE4 − group than in APOE4+. In addition, our findings also support a previous study that showed a significant association between APOE2, age, and β-amyloid in adults without cognitive impairment, in which the presence of the ε2 allele in APOE4+ individuals demonstrated a neuroprotective effect (Insel et al., 2021).

### 4.3. The sex-APOE4 interaction

The sex-APOE4 interaction is far from being clear, judging from current literature. The incidence of AD is reportedly higher in females than males (Farrer et al., 1997; Laws et al., 2018), especially in females aged 65 to 75 years old (Neu et al., 2017). Moreover, females with at least one copy of the APOE ε4 allele were found to have a twice greater risk of developing AD than male carriers (Genin et al., 2011; Hsu et al., 2019). In addition, females carrying the APOE4 allele are also reported to exhibit greater cognitive impairment, especially in memory, compared to male carriers (Ungar et al., 2014; Hsu et al., 2019). On the other hand, other studies reported no significant sex difference in the risk of developing mild cognitive impairment (MCI) and/or AD in older participants (aged between 55 and 85 years) that were carrying the APOE ε3/ε4 genotype (Neu et al., 2017). Moreover, Farrer et al. (1997) further indicated that this sex difference may decrease after the age of 75.

In our study, we found FA, MO, and NA were significantly greater in males than females across numerous WM regions, most notably in the corona radiata and internal capsule (Figures 4, 5). These areas are consistent with those related to the risk of developing AD (Serra et al., 2010; Yin et al., 2015; Mayo et al., 2018). However, the reduced anisotropy in women such as shown here do not necessarily indicate poorer WM health (Chad et al., 2021). In fact, in cross-fiber regions such as the internal capsule, enhanced anisotropy may be an indicator of selective fiber degeneration (Chad et al., 2021). Moreover, a recent study showed that physical activity was more beneficial in terms of reducing male APOE4 carriers’ brain age than for those of women carriers (Subramaniapillai et al., 2021). Thus, our results suggest that APOE4+ men exhibit greater degrees of WM degeneration than age-matched women. Moreover, the DT-DOME metrics of anisotropy (NA, MO) exhibit this sex difference much more strongly than FA. This can be attributed to DT-DOME metrics being more sensitive in these cross-fiber rich regions. These differences are almost identical to the sex differences between carrier-non-carrier groups combined (Figure 4), clearly indicating that the sex differences seen in cognitively normal APOE4− carriers extend into the APOE4+ group.

Beyond sex itself, our sample contained more ε4/ε4 and ε3/ε4 carrier males than females. Given that ε3 is a neutral allele, we may expect a genetically matched female carrier group (equal ε4/ε4 as in the male group) to exhibit even lower isotropy metrics than in the current sample. This subtle difference in group composition may also contribute to the greater sex differences in WM health amongst carriers, although there were no significant differences in sex ratios between the APOE4+ and APOE4− groups. This potential contribution remains to be confirmed in future research.

### 4.4. Conventional vs. novel DTI metrics

As mentioned earlier, many of the implicated regions in this study are crossing-fiber regions. In the absence of WM fiber crossings, conventional metrics derived from the tensor model are assumed to be based on its principal diffusion direction being aligned with the main orientation of all axonal fibers within the voxel. However, approximately 52% (Schilling et al., 2017) to 90% (Jeurissen et al., 2013) of all WM voxels contain crossing fibers, and so FA does not necessarily reflect microstructural integrity accurately in these voxels (Alba-Ferrara and de Erausquin, 2013). FA as a metric of anisotropy is actually confounded by MD (Ennis and Kindlmann, 2006; Chad et al., 2021). Thus, a lower FA is more likely to reflect age-related degeneration of single WM tracts than in voxels of crossing fibers (Cox et al., 2016).

In our previous work, we have demonstrated that the orthogonal-tensor decomposition (DT-DOME) yields DTI tensor-shape metrics (including MO and NA) that are unbiased by MD (Chad et al., 2021). NA provides a measure of the anisotropy of the fiber architecture, whereas MO provides a measure of the linearity of the fiber architecture (that is, MO can distinguish planar or “pancake-like” anisotropy from linear or “fiber-like” anisotropy). Thus, in this study, we assessed the sensitivity of these novel DTI metrics to the effect of APOE4, age, and sex on WM changes in regions of complex fiber architecture. Work by Cox et al., 2016 already revealed that MO is associated both positively and negatively with age across tracts in healthy older adults, depending on whether the tract was made up of voxels that included other crossing tracts. This is consistent with our own findings of both positive and negative age-association with MO and NA in the APOE4− group in the internal capsule (Figures 1, 2), a densely packed WM structure that runs at the inferomedial portion of each cerebral hemisphere and that contains overlap between projection and association fibers, which, respectively form the primary (from projection fibers) and secondary tract (from association fibers; (Emos and Agarwal, 2020). Based on our previous work, we interpret the increased MO and NA in aging as suggesting selective degeneration of a secondary tract, found primarily in regions of crossing fibers, such as the splenium of corpus callosum (crossing with posterior corona radiata), the superior longitudinal fasciculus (crossing with a number of commissural and projection fibers, including the corpus callosum and corticospinal tract), the internal capsule (crossing with fornix, corpus callosum and long association fibers), and the superior corona radiata (crossing with the body of corpus callosum and corticospinal tract). Both MO and NA showed these effects of possible selective degeneration more strongly than did FA among the APOE4− group. This is as expected, since MO and NA are completely independent of the increased extra-fiber isotropic diffusivity and, therefore, more specific to the fiber architecture. On the contrary, the finding that NA and MO are not positively correlated with age in the APOE4+ group was rather unexpected, suggesting that the selective degeneration of secondary crossing fibers that occurs in normal aging does not occur among APOE4 carriers. This finding may in fact be related to recruitment bias in the APOE+ group, as discussed earlier, rather than to the effect of APOE4 itself.

At the ROI level, MO was also significantly higher in the APOE4+ group in the fornix (crossing with the corpus callosum and internal capsule), while the conventional metrics did not show this difference (see Table 1). NA revealed yet more positive age associations than MO in the APOE4− group, including in the body of the callosum (cross with the corona radiata) and the external capsule. Positive age associations of NA without corresponding positive age associations of MO suggest simultaneous alterations in multiple fiber tracts, rather than selective degeneration of only the secondary tract (Chad et al., 2021). On the other hand, overlapping decreases in MO (less linear diffusion) and decreases in NA (less anisotropic diffusion irrespective of elevated MD) (Figure 4B) are found in regions with comparatively low architectural complexity, where the reduction in diffusion linearity and anisotropy may be due to demyelination. There is an almost perfect overlap amongst the negative age associations of FA, NA, and MO in such regions. Moreover, as discussed earlier, the stronger negative age associations of FA are likely attributable to the confounding effect of rising isotropic diffusion in aging. These trends all coincide with previous observations of age-related degeneration in a recent study by Chad et al. (2021).

Thus, our findings emphasize the importance of using DTI metrics independent of MD (MO and NA) in both the voxel-wise and ROI analyses of APOE4, age, and sex effects, especially in regions of more complex fiber architecture. The age- and sex-related differences in tissue anisotropy would not be as apparent if FA alone was used. In fact, our findings extend the evidence of selective degeneration, previously observed in healthy aging and AD, now to sex-related WM differences as well.

### 4.5. Strength, limitations, and future directions

This study has several strengths. First, our APOE4+ and APOE4− groups were matched in age, sex, education, and MMSE scores. Nevertheless, we found that sex differences may have driven the carrier versus non-carrier group differences in DTI-age associations, raising the important point that matching groups for demographic variables does not necessarily account for gene-expression differences between sexes. Second, another novel translational finding is the superior sensitivity of novel DT-DOME metrics to age-related and sex-related effects that may well be invisible to FA in regions of complex fiber architectures. Moreover, while the link between APOE4 and age-related effects is strong and widely established, the role of sex differences is commonly overlooked–a knowledge gap addressed directly by our study. We revealed consistent sex differences in both carrier and non-carrier groups particularly in the crossing fiber areas, e.g., internal capsule and corona radiata. Thus, the role of APOE4 carrier status on WM integrity can also be driven by sex differences, and future studies should include sex in their analyses.

One limitation of our study is the modest sample size. While we had access to a larger number of OASIS3 participants, we were restricted by our need for a single scanner platform, a short time from study entrance, and cognitively normal status. Having a single *b* = 0 volume in the diffusion MR acquisition and not being able to undergo TOP-UP correction are also limitations in this study, which could affect the data quality and could lead to misregistration. Going forward, enhanced preprocessing could enhance our ability to detect differences between APOE4+ and APOE4− groups. Moreover, as mentioned earlier, APOE protein level information is not provided in the OASIS-3 dataset, which might have limited the power of our investigation due to heterogeneity in APOE expression. Differences in the structure of each APOE isoform result in different physiological effects which can lead to differential susceptibility to disease. Thus, studies with larger cohorts that can select participants with more equal APOE isoform distributions are recommended to minimize the high inter-subject heterogeneity of the APOE genotypes. Finally, although our key finding was regarding the comparison across groups, we acknowledge that longitudinal studies are the gold standard for assessing age associations in DTI metrics of WM microstructure.

## 5. Conclusion

Our findings emphasize the influence of sex on the effects of APOE4 status on age-related differences in WM microstructure in cognitively normal older adults. DTI metrics independent of MD (MO and NA) may be more sensitive to group differences in age-related effects than conventional DTI metrics, especially in the regions of complex fiber architecture.

## Data availability statement

The original contributions presented in this study are included in the article/Supplementary material, further inquiries can be directed to the corresponding author.

## Ethics statement

According to provided information from the OASIS-3 Project (LaMontagne et al., 2019), human participants were reviewed and approved by the Institutional Review Board of Washington University School of Medicine. The patients/participants provided their written informed consent to participate in this study.

## Author contributions

PS, NA, and JJC contributed to the conception, design of the study, and methodology. PS wrote the draft of the manuscript, organized database, data recruitment and performed imaging, and statistical analyses. JAC contributed to the conception and methodology. JJC and PM provided funding. NA and JJC provided all analysis software. All authors contributed to the data visualization, manuscript revision, read, and approved the submitted version.

## Footnotes

1
https://www.oasis-brains.org

2
https://www.mrtrix.org/

3
https://fsl.fmrib.ox.ac.uk/fsl/fslwiki/GLM

## References

[B1] Abondio P. Sazzini M. Garagnani P. Boattini A. Monti D. Franceschi C. (2019). The genetic variability of APOE in different human populations and its implications for longevity. *Genes* 10:3. 10.3390/genes10030222 30884759 PMC6471373

[B2] Adluru N. Destiche D. J. Lu S. Y. Doran S. T. Birdsill A. C. Melah K. E. (2014). White matter microstructure in late middle-age: Effects of apolipoprotein E4 and parental family history of Alzheimer’s disease. *NeuroImage Clin.* 4 730–742. 10.1016/j.nicl.2014.04.008 24936424 PMC4053649

[B3] Alba-Ferrara L. M. de Erausquin G. A. (2013). What does anisotropy measure? Insights from increased and decreased anisotropy in selective fiber tracts in schizophrenia. *Front. Integr. Neurosci.* 7:9. 10.3389/fnint.2013.00009 23483798 PMC3593197

[B4] Altmann A. Tian L. Henderson V. Greicius M. Alzheimer’s Disease Neuroimaging Initiative Investigators (2014). Sex modifies the APOE-related risk of developing Alzheimer disease. *Ann. Neurol.* 75 563–573. 10.1002/ana.24135 24623176 PMC4117990

[B5] Basser P. J. Pajevic S. Pierpaoli C. Duda J. Aldroubi A. (2000). In vivo fiber tractography using DT-MRI data. *Magn. Reson. Med.* 44 625–632. 11025519 10.1002/1522-2594(200010)44:4<625::aid-mrm17>3.0.co;2-o

[B6] Behrens T. E. Woolrich M. W. Jenkinson M. Johansen-Berg H. Nunes R. G. Clare S. (2003). Characterization and propagation of uncertainty in diffusion-weighted MR imaging. *Magn. Reson. Med.* 50 1077–1088. 10.1002/mrm.10609 14587019

[B7] Benjamini Y. Hochberg Y. (1995). Controlling the false discovery rate: A practical and powerful approach to multiple testing. *J. R. Stat. Soc. Series B Stat. Methodol.* 57 289–300. 10.1111/j.2517-6161.1995.tb02031.x

[B8] Beydoun M. A. Boueiz A. Abougergi M. S. Kitner-Triolo M. H. Beydoun H. A. Resnick S. M. (2012). Sex differences in the association of the apolipoprotein E epsilon 4 allele with incidence of dementia, cognitive impairment, and decline. *Neurobiol. Aging* 33 720–731.e4. 10.1016/j.neurobiolaging.2010.05.017 20619505 PMC2974952

[B9] Bondi M. Houston W. Eyler T. Brown G. (2005). fMRI evidence of compensatory mechanisms in older adults at genetic risk for Alzheimer disease. *Neurology* 64 501–508. 10.1212/01.WNL.0000150885.00929.7E 15699382 PMC1761695

[B10] Bretsky P. M. Buckwalter J. G. Seeman T. E. Miller C. A. Poirier J. Schellenberg G. D. (1999). Evidence for an interaction between apolipoprotein E genotype, gender, and Alzheimer disease. *Alzheimer Dis. Assoc. Disord.* 13 216–221. 10.1097/00002093-199910000-00007 10609670

[B11] Cai S. Jiang Y. Wang Y. Wu X. Ren J. Lee M. S. (2017). Modulation on brain gray matter activity and white matter integrity by APOE ε4 risk gene in cognitively intact elderly: A multimodal neuroimaging study. *Behav. Brain Res.* 322 100–109. 10.1016/j.bbr.2017.01.027 28108320

[B12] Cavedo E. Lista S. Rojkova K. Chiesa P. A. Houot M. Brueggen K. (2017). Disrupted white matter structural networks in healthy older adult APOE ε4 carriers–An international multicenter DTI study. *Neuroscience* 357 119–133. 10.1016/j.neuroscience.2017.05.048 28596117

[B13] Chad J. A. Pasternak O. Chen J. J. (2021). Orthogonal moment diffusion tensor decomposition reveals age-related degeneration patterns in complex fiber architecture. *Neurobiol. Aging* 101 150–159. 10.1016/j.neurobiolaging.2020.12.020 33610963 PMC10902820

[B14] Cosentino S. Scarmeas N. Helzner E. Glymour M. M. Brandt J. Albert M. (2008). APOE ε4 allele predicts faster cognitive decline in mild Alzheimer disease. *Neurology* 70 1842–1849. 10.1212/01.wnl.0000304038.37421.cc 18401023 PMC2676693

[B15] Cox S. R. Ritchie S. J. Tucker-Drob E. M. Liewald D. C. Hagenaars S. P. Davies G. (2016). Ageing and brain white matter structure in 3,513 UK Biobank participants. *Nat. Commun.* 7:13629. 10.1038/ncomms13629 27976682 PMC5172385

[B16] Creavin S. Wisniewski S. Noel-Storr A. Trevelyan C. Hampton T. Rayment D. (2016). Mini-Mental State Examination (MMSE) for the detection of dementia in clinically unevaluated people aged 65 and over in community and primary care populations. *Cochrane Database Syst. Rev.* 2016:CD011145. 10.1002/14651858.CD011145.pub2 26760674 PMC8812342

[B17] de Groot M. Cremers L. Ikram M. Hofman A. Krestin G. van der Lugt A. (2016). White matter degeneration with aging: Longitudinal diffusion MR imaging analysis. *Radiology* 279 532–541. 10.1148/radiol.2015150103 26536311

[B18] Dell’Acqua F. Khan W. Gottlieb N. Giampietro V. Ginestet C. Bouls D. (2015). Tract based spatial statistic reveals no differences in white matter microstructural organization between carriers and non-carriers of the APOE ϵ4 and ϵ2 alleles in young healthy adolescents. *J. Alzheimers Dis.* 47 977–984. 10.3233/JAD-140519 26401776

[B19] Dhollander T. Connelly A. (2016). “A novel iterative approach to reap the benefits of multi-tissue CSD from just single-shell (+b=0),” in *Proceedings of the 24th international society of magnetic resonance in medicine*, Singapore.

[B20] Emos M. C. Agarwal S. (2020). *Neuroanatomy, internal capsule.* Treasure Island, FL: StatPearls. 31194338

[B21] Ennis D. B. Kindlmann G. (2006). Orthogonal tensor invariants and the analysis of diffusion tensor magnetic resonance images. *Magn. Reson. Med.* 55 136–146. 10.1002/mrm.20741 16342267

[B22] Farrer L. A. Cupples L. A. Haines J. L. Hyman B. Kukull W. A. Mayeux R. (1997). Effects of age, sex, and ethnicity on the association between apolipoprotein E genotype and Alzheimer disease. A meta-analysis. APOE and Alzheimer Disease Meta Analysis Consortium. *JAMA* 278 1349–1356. 10.1001/jama.1997.03550160069041 9343467

[B23] Fernandez C. G. Hamby M. McReynolds M. Ray W. (2019). The role of APOE4 in disrupting the homeostatic functions of astrocytes and microglia in aging and Alzheimer’s disease. *Front. Aging Neurosci.* 11:14. 10.3389/fnagi.2019.00014 30804776 PMC6378415

[B24] Fischl B. (2012). FreeSurfer. *NeuroImage* 62 774–781. 10.1016/j.neuroimage.2012.01.021 22248573 PMC3685476

[B25] Genin E. Hannequin D. Wallon D. Sleegers K. Hiltunen M. Combarros O. (2011). APOE and Alzheimer disease: A major gene with semi-dominant inheritance. *Mol. Psychiatry* 16 903–907. 10.1038/mp.2011.52 21556001 PMC3162068

[B26] Heise V. Filippini N. Ebmeier K. P. Mackay C. E. (2011). The APOE ϵ4 allele modulates brain white matter integrity in healthy adults. *Mol. Psychiatry* 16 908–916. 10.1038/mp.2010.90 20820167

[B27] Honea R. A. Vidoni E. Harsha A. Burns J. M. (2009). Impact of APOE on the healthy aging brain: A voxel-based MRI and DTI study. *J. Alzheimers Dis.* 18 553–564. 10.3233/JAD-2009-1163 19584447 PMC2892293

[B28] Hsu M. Dedhia M. Crusio W. E. Delprato A. (2019). Sex differences in gene expression patterns associated with the APOE4 allele. *F1000Research* 8:387. 10.12688/f1000research.18671.2 31448102 PMC6685458

[B29] Insel P. S. Hansson O. Mattsson-Carlgren N. (2021). Association between apolipoprotein E ε2 vs ε4, age, and β-amyloid in adults without cognitive impairment. *JAMA Neurol.* 78 229–235. 10.1001/jamaneurol.2020.3780 33044487 PMC7551211

[B30] Jeurissen B. Leemans A. Tournier J. Jones D. K. Sijbers J. (2013). Investigating the prevalence of complex fiber configurations in white matter tissue with diffusion magnetic resonance imaging. *Hum. Brain Mapp.* 34 2747–2766. 10.1002/hbm.22099 22611035 PMC6870534

[B31] Kaufer D. I. Cummings J. L. Ketchel P. Smith V. MacMillan A. Shelley T. (2000). Validation of the NPI-Q, a brief clinical form of the Neuropsychiatric Inventory. *J. Neuropsychiatry Clin. Neurosci.* 12 233–239. 10.1176/jnp.12.2.233 11001602

[B32] Kivipelto M. Solomon A. Ahtiluoto S. Ngandu T. Lehtisalo J. Antikainen R. (2013). The Finnish Geriatric Intervention Study to Prevent Cognitive Impairment and Disability (FINGER): Study design and progress. *Alzheimers Dement.* 9 657–665. 10.1016/j.jalz.2012.09.012 23332672

[B33] LaMontagne P. J. Benzinger T. Morris J. Keefe S. Hornbeck R. Xiong C. (2019). OASIS-3: Longitudinal neuroimaging, clinical, and cognitive dataset for normal aging and Alzheimer disease. *MedRxiv* [Preprint] 10.1101/2019.12.13.19014902

[B34] Laws K. R. Irvine K. Gale T. M. (2018). Sex differences in Alzheimer’s disease. *Curr. Opin. Psychiatry* 31 133–139. 10.1097/YCO.0000000000000401 29324460

[B35] Liu Y. Yu J. Wang H. Han P. Tan C. Wang C. (2015). APOE genotype and neuroimaging markers of Alzheimer’s disease: Systematic review and meta-analysis. *J. Neurol. Neurosurg. Psychiatry* 86 127–134. 10.1136/jnnp-2014-307719 24838911 PMC4331076

[B36] Liu Z. Zhu H. Marks B. Katz L. Goodlett C. Gerig G. (2009). “Voxel-wise group analysis of DTI,” in *Proceedings of the IEEE international symposium on biomedical imaging: From nano to macro*, (Boston, MA), 807–810. 10.1109/isbi.2009.5193172 PMC3660096 23703686

[B37] Martins C. Oulhaj A. de Jager C. Williams J. (2005). APOE alleles predict the rate of cognitive decline in Alzheimer disease: A nonlinear model. *Neurology* 65 1888–1893. 10.1212/01.wnl.0000188871.74093.12 16380608

[B38] Mayo C. D. Garcia-Barrera M. A. Mazerolle E. L. Ritchie L. J. Fisk J. D. Gawryluk J. R. (2018). Relationship between DTI metrics and cognitive function in Alzheimer’s disease. *Front. Aging Neurosci.* 10:436. 10.3389/fnagi.2018.00436 30687081 PMC6333848

[B39] Mori S. Oishi K. Jiang H. Jiang L. Li X. Akhter K. (2008). Stereotaxic white matter atlas based on diffusion tensor imaging in an ICBM template. *NeuroImage* 40 570–582. 10.1016/j.neuroimage.2007.12.035 18255316 PMC2478641

[B40] Morris J. C. (1993). The clinical dementia rating (CDR): Current version and scoring rules. *Neurology* 43 2412–2414. 10.1212/WNL.43.11.2412-a 8232972

[B41] Mortensen E. L. Høgh P. (2001). A gender difference in the association between APOE genotype and age-related cognitive decline. *Neurology* 57 89–95. 10.1212/wnl.57.1.89 11445633

[B42] Neu S. C. Pa J. Kukull W. Beekly D. Kuzma A. Gangadharan P. (2017). Apolipoprotein E genotype and sex risk factors for Alzheimer disease: A meta-analysis. *JAMA Neurol.* 74 1178–1189. 10.1001/jamaneurol.2017.2188 28846757 PMC5759346

[B43] Nichols T. E. Holmes A. P. (2002). Nonparametric permutation tests for functional neuroimaging: A primer with examples. *Hum. Brain Mapp.* 15 1–25. 10.1002/hbm.1058 11747097 PMC6871862

[B44] Nierenberg J. Pomara N. Hoptman M. J. Sidtis J. J. Ardekani B. A. Lim K. O. (2005). Abnormal white matter integrity in healthy apolipoprotein E epsilon4 carriers. *Neuroreport* 16 1369–1372. 10.1097/01.wnr.0000174058.49521.16 16056141

[B45] Operto G. Cacciaglia R. Grau-Rivera O. Falcon C. Brugulat-Serrat A. Ródenas P. (2018). White matter microstructure is altered in cognitively normal middle-aged APOE-ε4 homozygotes. *Alzheimers Res. Ther.* 10:48. 10.1186/s13195-018-0375-x 29793545 PMC5968505

[B46] Paraskevaidi M. Morais C. L. Lima K. M. Snowden J. S. Saxon J. A. Richardson A. M. (2017). Differential diagnosis of Alzheimer’s disease using spectrochemical analysis of blood. *Proc. Natl. Acad. Sci. U.S.A.* 114 E7929–E7938. 10.1073/pnas.1701517114 28874525 PMC5617251

[B47] Persson J. Lind J. Larsson A. Ingvar M. Cruts M. Broeckhoven C. V. (2006). Altered brain white matter integrity in healthy carriers of the APOE epsilon4 allele: A risk for AD? *Neurology* 66 1029–1033. 16606914 10.1212/01.wnl.0000204180.25361.48

[B48] Pfeffer R. I. Kurosaki T. T. Harrah C. Jr. Chance J. M. Filos S. (1982). Measurement of functional activities in older adults in the community. *J. Gerontol.* 37 323–329. 10.1093/geronj/37.3.323 7069156

[B49] Ryan L. Walther K. Bendlin B. B. Lue L. Walker D. G. Glisky E. L. (2011). Age-related differences in white matter integrity and cognitive function are related to APOE status. *NeuroImage* 54 1565–1577. 10.1016/j.neuroimage.2010.08.052 20804847 PMC2997188

[B50] Saddiki H. Fayosse A. Cognat E. Sabia S. Engelborghs S. Wallon D. (2020). Age and the association between apolipoprotein E genotype and Alzheimer disease: A cerebrospinal fluid biomarker-based case-control study. *PLoS Med.* 17:e1003289. 10.1371/journal.pmed.1003289 32817639 PMC7446786

[B51] Salminen L. E. Schofield P. R. Lane E. M. Heaps J. M. Pierce K. D. Cabeen R. (2013). Neuronal fiber bundle lengths in healthy adult carriers of the ApoE4 allele: A quantitative tractography DTI study. *Brain Imaging Behav.* 7 274–281. 10.1007/s11682-013-9225-4 23475756 PMC3726531

[B52] Schilling K. Gao Y. Janve V. Stepniewska I. Landman B. A. Anderson A. W. (2017). Can increased spatial resolution solve the crossing fiber problem for diffusion MRI? *NMR Biomed.* 30:12. 10.1002/nbm.3787 28915311 PMC5685916

[B53] Serra L. Cercignani M. Lenzi D. Perri R. Fadda L. Caltagirone C. (2010). Grey and white matter changes at different stages of Alzheimer’s disease. *J. Alzheimers. Dis.* 19 147–159. 10.3233/JAD-2010-1223 20061634

[B54] Sheikh J. I. Yesavage J. A. (1986). Geriatric Depression Scale (GDS): Recent evidence and development of a shorter version. *Clin. Gerontol.* 5 165–173. 10.1300/J018v05n01_09

[B55] Slattery C. F. Zhang J. Paterson R. W. Foulkes A. J. Carton A. Macpherson K. (2017). ApoE influences regional white-matter axonal density loss in Alzheimer’s disease. *Neurobiol. Aging* 57 8–17. 10.1016/j.neurobiolaging.2017.04.021 28578156 PMC5538347

[B56] Smith S. M. (2002). Fast robust automated brain extraction. *Hum. Brain Mapp.* 17 143–155. 10.1002/hbm.10062 12391568 PMC6871816

[B57] Smith S. Jenkinson M. Johansen-Berg H. Rueckert D. Nichols T. Mackay C. (2006). Tract-based spatial statistics: Voxelwise analysis of multi-subject diffusion data. *NeuroImage* 31 1487–1505. 10.1016/j.neuroimage.2006.02.024 16624579

[B58] Solomon A. Turunen H. Ngandu T. Peltonen M. Levälahti E. Helisalmi S. (2018). Effect of the apolipoprotein E genotype on cognitive change during a multidomain lifestyle intervention: A subgroup analysis of a randomized clinical trial. *JAMA Neurol.* 75 462–470. 10.1001/jamaneurol.2017.4365 29356827 PMC5885273

[B59] Storey J. D. (2002). A direct approach to false discovery rates. *J. R. Stat. Soc. Series B Stat. Methodol.* 64 479–498. 10.1111/1467-9868.00346

[B60] Subramaniapillai S. Rajagopal S. Snytte J. Otto A. R. Group Prevent-Ad Research, Einstein G. (2021). Sex differences in brain aging among adults with family history of Alzheimer’s disease and APOE4 genetic risk. *NeuroImage Clin.* 30:102620. 10.1016/j.nicl.2021.102620 33857772 PMC8065341

[B61] Tournier J.-D. Smith R. Raffelt D. Tabbara R. Dhollander T. Pietsch M. (2019). MRtrix3: A fast, flexible and open software framework for medical image processing and visualisation. *NeuroImage* 202:116137. 10.1016/j.neuroimage.2019.116137 31473352

[B62] Ungar L. Altmann A. Greicius M. D. (2014). Apolipoprotein E, gender, and Alzheimer’s disease: An overlooked, but potent and promising interaction. *Brain Imaging Behav.* 8 262–273. 10.1007/s11682-013-9272-x 24293121 PMC4282773

[B63] Wakana S. Jiang H. Nagae-Poetscher L. van Zijl P. Mori S. (2004). Fiber tract-based atlas of human white matter anatomy. *Radiology* 230 77–87. 10.1148/radiol.2301021640 14645885

[B64] Wang R. Fratiglioni L. Laukka E. J. Lövdén M. Kalpouzos G. Keller L. (2015). Effects of vascular risk factors and APOE ε4 on white matter integrity and cognitive decline. *Neurology* 84 1128–1135. 10.1212/WNL.0000000000001379 25672924 PMC4371409

[B65] Winkler A. M. Ridgway G. R. Webster M. A. Smith S. M. Nichols T. E. (2014). Permutation inference for the general linear model. *NeuroImage* 92 381–397. 10.1016/j.neuroimage.2014.01.060 24530839 PMC4010955

[B66] Yin R. Tan L. Liu Y. Wang W. Wang H. Jiang T. (2015). Multimodal voxel-based meta-analysis of white matter abnormalities in Alzheimer’s disease. *J. Alzheimers Dis.* 47 495–507. 10.3233/JAD-150139 26401571 PMC5757541

[B67] Zhao L. Wu L. (2016). ApoE2 and Alzheimer’s disease: Time to take a closer look. *Neural Regen. Res.* 11 412–413. 10.4103/1673-5374.179044 27127474 PMC4829000

