# Supplementary material for: Effect of sex on the APOE4-aging interaction in the white matter microstructure of cognitively normal older adults using diffusion-tensor MRI with orthogonal-tensor decomposition (DT-DOME)
Source: Front Neurosci. 2023 Feb 22;17:1049609. doi: 10.3389/fnins.2023.1049609 (PMC9992882; doi:10.3389/fnins.2023.1049609)
Supplement: Supplementary file 1 [file Data_Sheet_1.docx]

Supplementary Material

**Supplementary Table 1.** Summary of current literature on diffusion-MRI based differences between APOE4 carrier (APOE4+) and noncarrier (APOE4-) groups. The sex ratio is listed as the number of males versus females (M:F) and as the ratio (number of males per female). Note: fractional anisotropy, FA; mean diffusivity, MD; axial diffusivity, AxD; radial diffusivity, RD; mode of anisotropy, MO; and norm of anisotropy, NA; gray matter, GM; white matter, WM; parahippocampal gyrus, PHC; corpus callosum, CC; genu and splenium of the corpus callosum, gCC and sCC; cingulum, Cg; superior longitudinal fasciculus, SLF; inferior longitudinal fasciculus, ILF; inferior fronto-occipito fasciculus, IFOF; uncinate fasciculus, UF; fornix, Fx; corona radiata, CR; superior corona radiata, SCR; internal capsule, IC; external capsule, EC; anterior thalamic radiation, ATR; corticospinal tracts, CST; years old, y/o; Alzheimer’s Disease Neuroimaging Initiative database, ADNI; and National Alzheimer's Coordinating Center dataset, NACC.

| **Study** | **Sample Size (N)** | | **Age (y/o)**  **(mean±SD)** | **Findings** |
| --- | --- | --- | --- | --- |
|  | **APOE4+**  **(M:F)** | **APOE4-**  **(M:F)** |  |  |
| **Age- and Sex-related Differences in Healthy Aging** | | | | |
| [Honea et al., 2009](https://paperpile.com/c/HrFV4M/5E80) | 6:8  (0.75M:1F) | 24:15  (1.6M:1F) | 73.40±6.30 | - APOE4+ trended towards lower FA in Lt. PHC when compared to APOE4-.  - No significant differences between APOE4+ and APOE4- in the normalized whole brain, GM or WM volume.  - APOE4+ had significantly lower global cognition compared to the APOE4-. |
| [Adluru et al., 2014](https://paperpile.com/c/HrFV4M/n4IE) | 40:86  (0.46M:1F) | 74:144  (0.51M:1F) | 60.83±6.72 | - Effect of age was found in all DTI metrics, notably in Cg, CC, and Fx.  - No effects of the APOE4 were found.  - Effect of sex was only found in FA and RD, but not in MD and AxD.  - Males had lower FA in the sCC, Cg, SLF, and UF, and higher RD in the Cg, Fx, and SLF compared to females. |
| [Dell’Acqua et al., 2015](https://paperpile.com/c/HrFV4M/WAYQ) | 231:196  (1.18M:1F) | 70:49  (1.43M:1F) | 14.43±0.47 | - No significant difference in any DTI metrics between APOE4+ and APOE4-. |
| [Wang et al., 2015](https://paperpile.com/c/HrFV4M/E2TJ) | 24:49  (0.49M:1F) | 65:103  (0.63M:1F) | 72.00±9.00 | - No significant difference in any DTI metric between APOE4+ and APOE4-. |
| [Cai et al., 2017](https://paperpile.com/c/HrFV4M/K8P6) | 13:14  (0.93M:1F) | 14:17  (0.82M:1F) | 72.80±4.63 | - AxD and MD in the gCC was lower in APOE4 APOE4+ than APOE4-.  - MD in SCR was lower in APOE4+ than APOE4-.  - No significant difference in FA and RD between APOE4+ and APOE4-.  - No significant changes in GM and WM volume between APOE4+ and APOE4-. |
| [Cavedo et al., 2017](https://paperpile.com/c/HrFV4M/QlUy) | 15:16  (0.94M:1F) | 20:23  (0.97M:1F) | 68.95±6.85 | - Significantly increased RD and reduced FA in Cg, CC, and ILF in APOE4+ compared to APOE4-.  - APOE4+ showed higher MD in gCC, IC, EC, SLF, and CR compared to APOE4-. |
| [Operto et al., 2018](https://paperpile.com/c/HrFV4M/Csgk) | 119:156  (0.76M:1F) | 92:165  (0.56M:1F) | 58.13±7.46 | - Significantly increased MD in SLF in APOE4+ (e4e4), but no significant difference in FA.  - Effect of age was found in all DTI metrics, most notably in Cg, CC, SLF, ILF, and IFOF. |

**Supplementary Table 2.** Summary of current literature on sex and APOE4-related differences in aging and dementia. Where available, the sex ratio is listed in the number of males versus females (M:F) and as the ratio (number of males per female). Note: fractional anisotropy, FA; mean diffusivity, MD; axial diffusivity, AxD; radial diffusivity, RD; mode of anisotropy, MO; and norm of anisotropy, NA; gray matter, GM; white matter, WM; parahippocampal gyrus, PHC; corpus callosum, CC; genu and splenium of the corpus callosum, gCC and sCC; cingulum, Cg; superior longitudinal fasciculus, SLF; inferior longitudinal fasciculus, ILF; inferior fronto-occipito fasciculus, IFOF; uncinate fasciculus, UF; fornix, Fx; corona radiata, CR; superior corona radiata, SCR; internal capsule, IC; external capsule, EC; anterior thalamic radiation, ATR; corticospinal tracts, CST; years old, y/o; Alzheimer’s Disease Neuroimaging Initiative database, ADNI; and National Alzheimer's Coordinating Center dataset, NACC.

| **Study** | **Sample Size (N)** | | **Age (y/o)**  **(mean±SD)** | **Findings** |
| --- | --- | --- | --- | --- |
|  | **APOE4+**  **(M:F)** | **APOE4-**  **(M:F)** |  |  |
| **Sex Differences in Clinical Progression in Healthy Aging** | | | | |
| [Altmann et al., 2014](https://paperpile.com/c/HrFV4M/a5Zs)   - NACC - ADNI | 535:1,060  (0.50M:1F)  37:34  (1.09M:1F) | 1,309:2,592  (0.51M:1F)  100:101  (0.99M:1F) | 73 (66.6-79.8)  73.7 (70.8-78.11) | - Significant sex-APOE4 interaction in healthy older adults.  - Both male and female APOE4+ were at the higher risk for clinical progression (controls to MCI/AD), while the APOE4- males were at intermediate risk, and noncarrier females are at the least risk in progressing from controls to MCI/AD.  - Healthy older female APOE4+ had significantly higher conversion rate to MCI or AD than older female APOE4-.  - Healthy older males APOE4+ had a marginally significant increase in conversion risk to MCI or AD compared to males APOE4-. |
|  |  |  |  |  |
|  |  |  |  |  |
| [Bretsky et al., 1999](https://paperpile.com/c/HrFV4M/zcxo) | 19:22  (0.86M:1F) | 17:57  (0.30M:1F) | 76.2±8.0 | - Females carrying one or more ε4 had a significantly increased AD risk than males carrying one or more ε4.  - Males carrying one or more ε4 did not have significantly increased AD risk. |
| [Beydoun et al., 2012](https://paperpile.com/c/HrFV4M/WxbR) | 88:84  (1.05M:1F) | 286:186  (1.54M:1F) | 55.53±15.25  (17-97) | - Female APOE4+ had a significantly higher incidence of dementia than female APOE4-, but not in male.  - Male APOE4+ do not have a significantly higher incidence of dementia than male APOE4-.  - Females have a higher chance to carry the ε4 allele than males. |
| [Mortensen and Høgh, 2001](https://paperpile.com/c/HrFV4M/g6iY) | 26:26  (1M:1F) | 53:58  (0.91M:1F) | 50-80 | - A significant interaction between sex and APOE genotypes was observed.  - Females carrying ε4 showed significantly more decline in IQ performance than female APOE4-.  - Male APOE4- showed more decline in IQ than male APOE4+.  - The impact of the APOE4 on cognitive decline in females was observed among 70 to 80 year olds. |
| **Sex-Related Effect in MCI, AD, and Controls** | | | | |
| [Farrer et al., 1997](https://paperpile.com/c/HrFV4M/5JL6)  Between AD and controls | Meta-analysis: 40 studies  ε4+ : ε4- = 5,403:9,075  M:F = 5,732:8,746  No sex-specific APOE+ and APOE- numbers given | | 40-90 | - Age-related changes toward the development of Alzheimer’s disease were more prominent in homozygous than heterozygous ε4 carriers in both sexes.  - Significant interaction between sex-APOE4 was found but not uniform across APOE genotypes. Females carrying ε4 were more likely to develop AD than males.  - Both APOE effect and sex difference were found to diminish after age 75. |
| [Neu et al., 2017](https://paperpile.com/c/HrFV4M/zGzd)  In Control/ MCI/AD | Meta-analysis: 27 studies  41,036 participants  I. Control  - ε4+ : ε4- = 4,696:11,054  - M:F = 6,023:9,727  II. MCI  - ε4+ : ε4- = 9772:5,941  - M:F = 5,289:4,284  III. AD  - ε4+ : ε4- = 4,103:5,470  - M:F = 7,323:8,390  No sex-specific APOE+ and APOE- numbers given | | 55-85 | - Females with ε4 carriers ages between 65 and 75 year old had an increased risk of developing AD than males.  - Males carriers had a higher risk of developing AD than the males noncarriers.  - No significant difference between homozygous ε4 carriers males and females was found. |
| [Payami et al., 1996](https://paperpile.com/c/HrFV4M/KUbs)  In late-onset familial AD | 85:131  (0.63M:1F) | 28:51  (0.55M:1F) | For females,  69.98±7.5  For males,  68.5±8.2 | - Females heterozygous ε4 carriers had higher risk for AD conversion than the APOE4-, and no significant difference in males.  - No significant difference between females hetero- and homogygous ε4 carriers, while males homozygous e4 carriers had significant higher risk to develop AD than those with heterozygous carriers. |

***Supplementary Figure Captions***

**Supplementary Figure 1. ROI-wise Pearson correlation coefficient (r) across age, education, MMSE, DTI and orthogonal-tensor decomposition metrics, spanning the APOE4+ and APOE4− groups.** The color bar shows the significant negative (marked in blue) and positive (marked in red) correlation at *p*<0.05 and *p*<0.01. All *p*-values were corrected for multiple comparisons based on false discovery rate (FDR) at *p*<0.05. Note: Education level, Edu; Mini-Mental State Exam score, MMSE; the whole tract of the fornix, Fx_w; the body and column of the fornix, Fx_BC; corpus callosum, CC; cingulum bundle, Cg; corona radiata, CR; posterior thalamic radiation, PTR; internal capsule, IC; external capsule, EC; superior longitudinal fasciculus, SLF; inferior longitudinal fasciculus, ILF; fractional anisotropy, FA; mean diffusivity, MD; axial diffusivity, AxD; radial diffusivity, RD; mode of anisotropy, MO; and norm of anisotropy, NA.

**Supplementary Figure 2. Grouped forest plot displaying the most prominent combinations of significant associations between DTI and orthogonal-tensor decomposition metrics and age among APOE4 carrier (APOE4+) and non-carrier (APOE4−) groups.** The overlapping regions that show significant negative age-associations in FA along with positive age-associations in MD, AxD, and RD were found in most WM tracts in both groups as shown in **(A)**. The overlapping regions that show significant negative age associations of both MO and NA in both groups were shown in **(B)**, and significant positive age associations of both MO and NA are found only in APOE4− as shown in **(C)**. (←) indicates negative age-association and (→) indicates positive age-association. Note: fractional anisotropy, FA; mean diffusivity, MD; axial diffusivity, AxD; radial diffusivity, RD; mode of anisotropy, MO; norm of anisotropy, NA; and region-of-interest, ROIs.

**Supplementary Figure 3. Grouped forest plot displaying the most prominent overlap and dissociations in significant age effects between APOE4 carrier (APOE4+) and non-carrier (APOE4−) groups.** The overlapping regions show the significant negative age associations of FA, MO, and NA in the APOE4+ **(A)** and APOE4− groups **(B and C)**. The overlapping regions that showed the significant positive age associations of age-related MO and NA, but not FA are shown in **(C)**. Note: fractional anisotropy, FA; mean diffusivity, MD; axial diffusivity, AxD; radial diffusivity, RD; mode of anisotropy, MO; norm of anisotropy, NA, region-of-interest, ROIs.
