# Supplementary figures and images for: Effect of sex on the APOE4-aging interaction in the white matter microstructure of cognitively normal older adults using diffusion-tensor MRI with orthogonal-tensor decomposition (DT-DOME)
Source: Front Neurosci. 2023 Feb 22;17:1049609. doi: 10.3389/fnins.2023.1049609 (PMC9992882; doi:10.3389/fnins.2023.1049609)

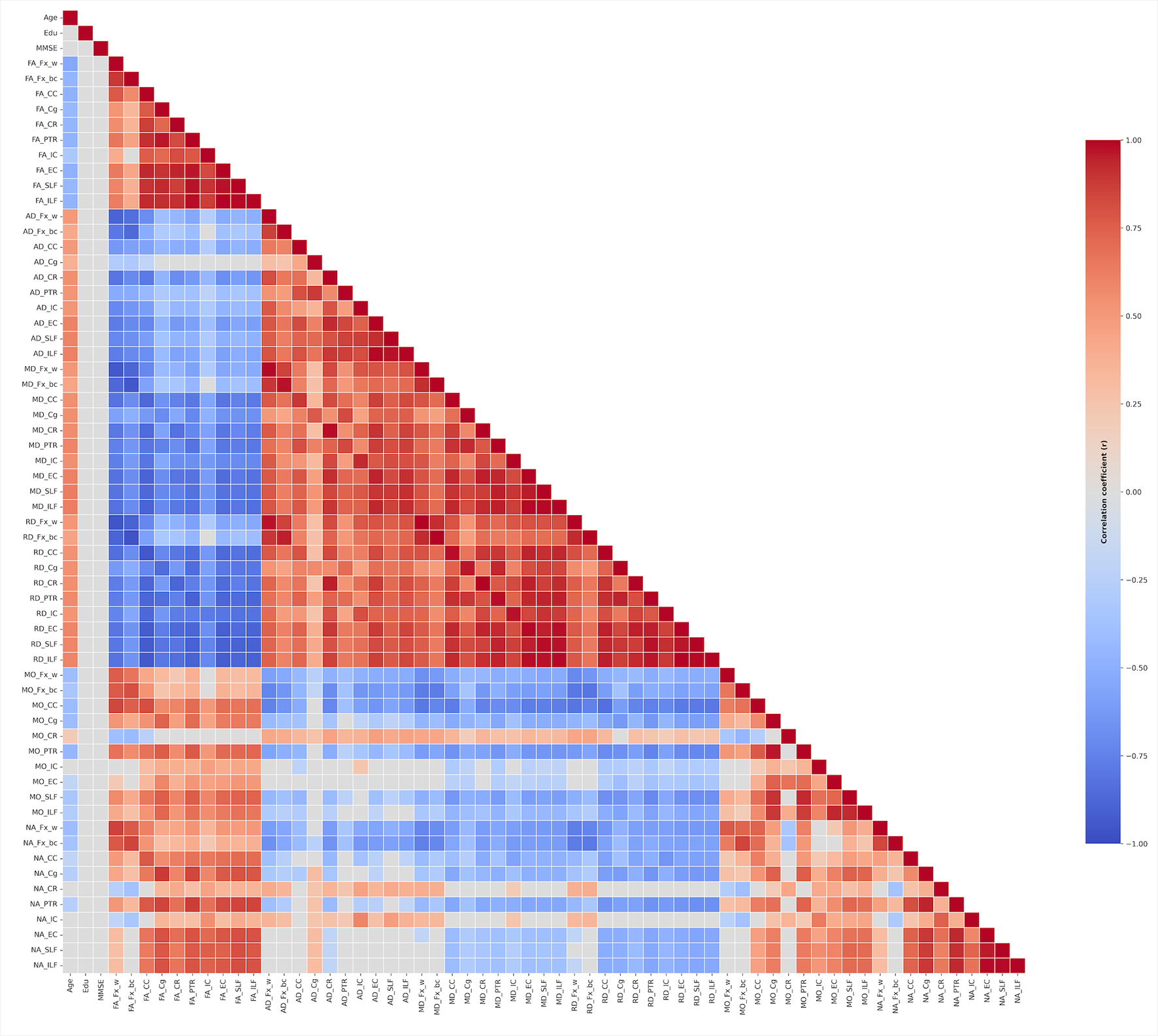

Supplement: Supplementary file 2 [file Image_1.tiff]

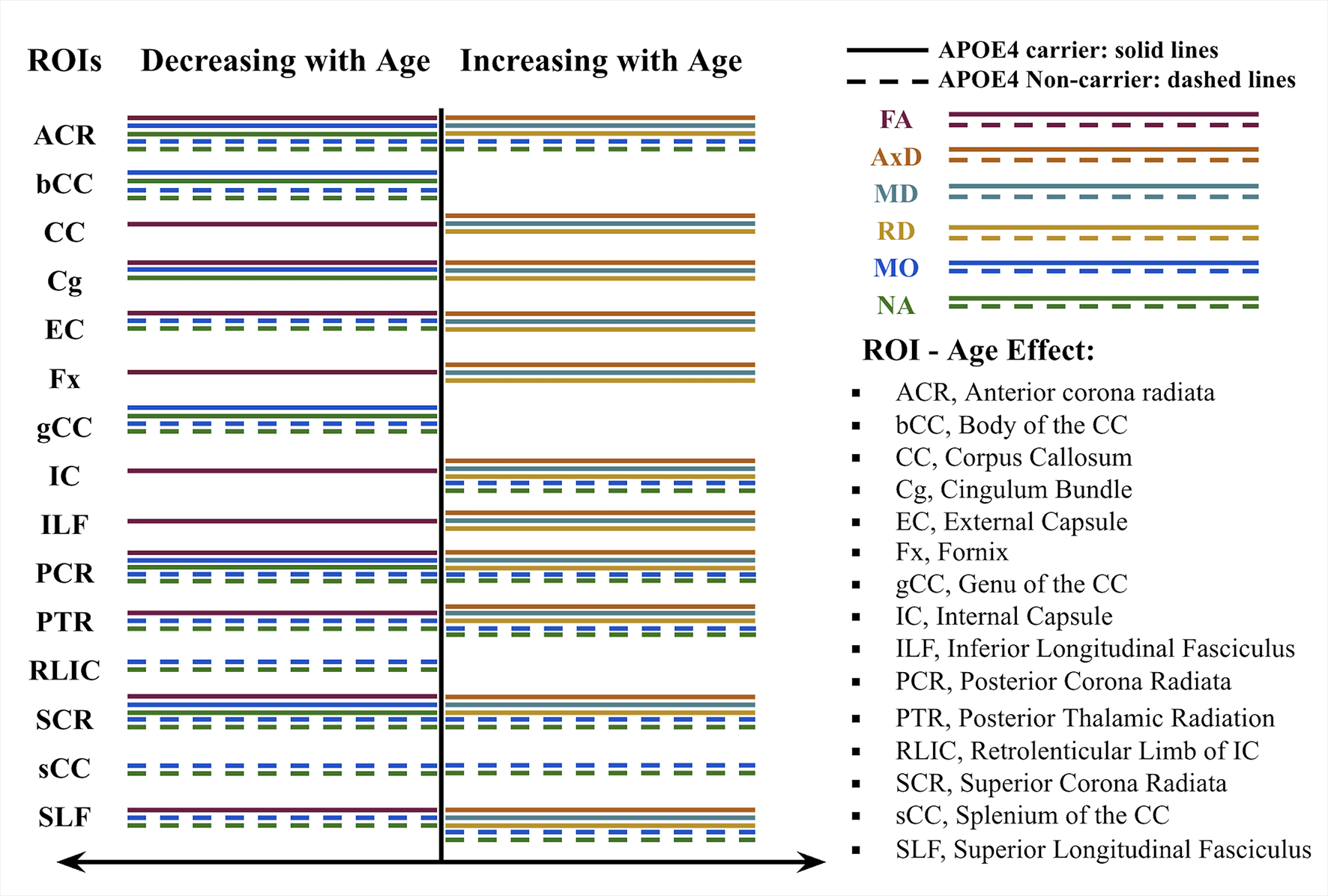

Supplement: Supplementary file 3 [file Image_2.tiff]

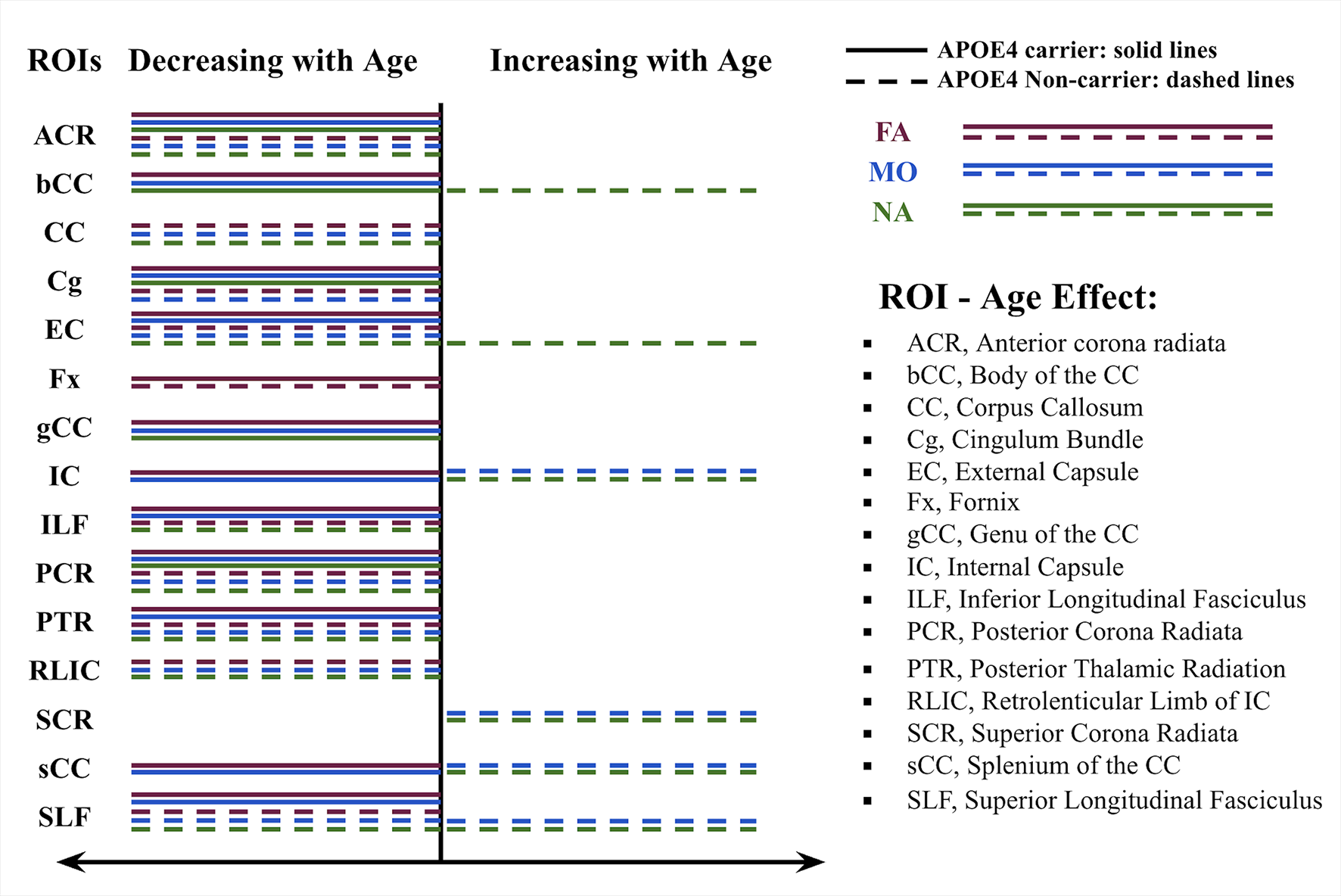

Supplement: Supplementary file 4 [file Image_3.tiff]
